# Perinatal caffeine administration improves outcomes in an ovine model of neonatal hypoxia-ischemia

**DOI:** 10.1161/STROKEAHA.124.048264

**Published:** 2024-10-21

**Authors:** Jana K Mike, Yasmine White, Janica Ha, Ariana Iranmahboub, Cheryl Hawkins, Rachel S Hutchings, Christian Vento, Hadiya Manzoor, Aijun Wang, Brian D Goudy, Payam Vali, Satyan Lakshminrusimha, Jogarao VS Gobburu, Janel Long-Boyle, Jeffrey R Fineman, Donna M Ferriero, Emin Maltepe

**Affiliations:** a Department of Pediatrics, University of California San Francisco, San Francisco, CA, USA; b Department of Biomedical Engineering, University of California Davis, Davis, CA, USA; c Department of Pediatrics, University of California Davis, Davis, CA, USA; d School of Pharmacy, University of Maryland, Baltimore, MD, USA; e Initiative for Pediatric Drug and Device Development, San Francisco, CA, USA; f School of Pharmacy, University of California San Francisco, San Francisco, CA, USA; g Department of Neurology, Weill Institute for Neurosciences, University of California San Francisco, San Francisco, CA, USA; h Department of Biomedical Sciences, University of California San Francisco, San Francisco, CA, USA

**Keywords:** ovine model, caffeine, brain hypoxia-ischemia, neonatal

## Abstract

**Background:**

Neonatal hypoxic-ischemic encephalopathy (HIE) disproportionately affects low- and middle-income countries (LMIC) where nearly 96 % of affected infants reside. The current standard of care, therapeutic hypothermia, is frequently ineffective in this setting, likely because injury may be occurring earlier during labor. Here, we studied the pharmacokinetics, safety, and efficacy of perinatal caffeine administration in near-term lambs following global ischemic injury to support the development of earlier treatment strategies targeting the fetus in utero as well as the infant postnatally.

**Methods:**

Ewes were randomly assigned to receive either 1 g intravenous (IV) caffeine citrate or placebo prior to delivery and placental transport assessed. Near-term lambs (141–143 days) of both sexes were subjected to severe global hypoxia-ischemia utilizing an acute umbilical cord occlusion (UCO) model. Lambs that received caffeine in utero also received 20 mg/kg IV caffeine citrate following resuscitation and 10 mg/kg/day IV for two days. An additional cohort received 60 mg/kg followed by 30 mg/kg/day (low dose vs high dose) postnatally. Biochemical, histological and neurological outcomes measures in lambs were assessed over a six-day period.

**Results:**

Perinatal caffeine administration demonstrated excellent placental transport kinetics and was well tolerated with lamb plasma levels comparable to those targeted in neonates with apnea of prematurity. Caffeine administration resulted in a systemic immunomodulatory effect evidenced by significant reductions in pro-inflammatory IP-10 levels. Treated lambs demonstrated improved neurodevelopmental outcomes while histological analysis revealed that caffeine reduced gray matter injury and attenuated inflammation in the cingulate and parasagittal cortex. This neuroprotective effect was greater and via a different mode of action than we previously reported for azithromycin. A higher caffeine dosing regimen demonstrated significant toxicity.

**Conclusion:**

Perinatal caffeine administration is well tolerated, attenuates systemic and brain inflammation, and contributes to improvements in histological and neurological outcomes in an ovine model of neonatal HIE.

## Graphical Abstract

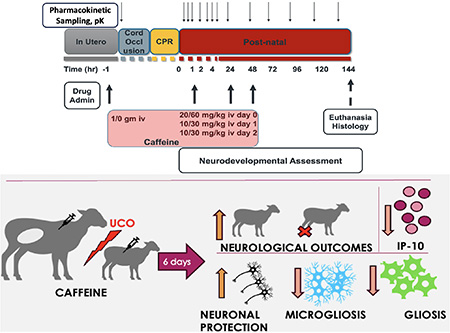

## 1. Introduction

Greater than 95% of global cases of cerebral palsy^1^ can be found in low- and middle-income countries (LMIC) and are frequently caused by neonatal brain injury/hypoxic-ischemic encephalopathy (HIE). Multiple factors contribute to a higher incidence of HIE in LMIC, including chronic placental insufficiency^2^. The current standard of care, therapeutic hypothermia (TH), is only modestly effective in high income countries (HIC), while its use in LMIC is frequently ineffective or contraindicated due to significantly increased adverse events^3^. The search for alternate neuroprotective therapies has been extensive^4,5^, yet many, including caffeine, have not been tested in clinical trials without TH. Additionally, some are limited by the requirement for early therapy or the lack of pharmaceutical grade commercial preparations, as well as safety concerns in the postnatal setting, such as with magnesium^6^. Importantly, the likely earlier timing of brain injury associated with HIE in LMIC^7^ is most likely due to chronic placental insufficiency resulting in greater growth restriction and intolerance of labor triggering repetitive hypoxic insults in utero^7,8^. This suggests that antenatal administration of agents with favorable placental transfer kinetics may be necessary to ensure maximal efficacy.

Caffeine citrate (caffeine) has demonstrated an excellent safety profile in preterm neonates over the past 40 years and is the standard of care treatment for apnea of prematurity^9^. The Caffeine for Apnea of Prematurity trial demonstrated reduced risks of cerebral palsy and cognitive delay at 18 months with a daily treatment regimen consisting of a 20 mg/kg loading dose followed by daily dosing of up to 10 mg/kg. The effects extended long-term, as benefits were noted on motor development at 5 years of age^10^ and visuomotor, visuoperceptual, and visuospatial abilities at 11 years^11^. The neuroprotective properties of caffeine have also been observed in vitro as well as in various animal models of HIE^9,12^.

Caffeine and other methylxanthines such as theophylline are primarily used to block A_1_ and A_2A_ adenosine receptors to help stimulate breathing, with predominant blockade of A_2A_ noted during brain ischemia^13^. Adenosine receptors play a dual role in brain ischemia, and antagonism may be beneficial in the brain during later stages, where prolonged activation in neurons may be detrimental^14^. Additionally, caffeine acts as a potent antioxidant^15^, and can support integrity of the blood-brain barrier^16^ while influencing inflammation locally as well as systemically^17^. At lower doses, caffeine is a competitive inhibitor of adenosine A_1_, A_2A_ and A_2B_ receptors, while at higher concentrations it can inhibit phosphodiesterase activity, which is responsible for many of its reported adverse effects^18^.

Preclinical models in sheep have been instrumental for advancing some of the most impactful therapies in maternal-fetal medicine and neonatology today. These include antenatal steroids, resuscitation approaches, surfactant, and therapeutic hypothermia^19,20^. Due to the potential need for in utero treatment approaches for HIE in the LMIC setting, the ability to define placental transport kinetics, fetal pharmacology, safety as well as postnatal efficacy of potential therapeutics requires physiologically relevant large animal models such as sheep to aid translational efforts. In this context, we evaluated the safety, pharmacokinetics, mode of action and neuroprotective efficacy of perinatal caffeine administration in our model of HIE in near-term lambs designed to develop therapies for LMIC. We additionally evaluated whether a higher dosing regimen than used clinically would be feasible.

## 2. Methods

### Data availability

The data presented in this work are available by reasonable request. Full descriptions of Methods are available in the Supplemental Material.

### Animals

All animal research was approved by the University of California Davis Institutional Animal Care and Use Committee and was performed in accordance with the Guide for the Care and Use of Laboratory Animals and follows ARRIVE 2.0 guidelines^21^.

### Neonatal hypoxia-ischemia

HIE was induced via UCO (umbilical cord occlusion) in near term lambs at 141–143 days gestation (term ~ 147–150 days) as previously described^2,22,23^. Physiological parameters, such as blood pressure, heart rate, the onset of return of spontaneous circulation (ROSC) and the epinephrine doses required for ROSC were assessed.

### Drug Treatment

Two caffeine dosing regimens were tested. In the first, ewes were randomized to receive either 1 g of intravenous (IV) caffeine citrate or placebo total prior to cesarean section. Following delivery, lambs born to ewes that received caffeine were administered 20 mg/kg IV caffeine citrate over 10 minutes starting 10 minutes following resuscitation, along with two additional doses of 10 mg/kg IV caffeine citrate each at 24 and 48 hours of life (Low Dose, LD). An additional high dose (HD) caffeine arm was also investigated where no caffeine was administered to the ewe, but each lamb was randomized to receive either placebo or 60 mg/kg IV caffeine citrate following resuscitation, and 30 mg/kg IV at 24 and 48 hours of life. For details, please see the Supplemental Material.

### Pharmacokinetic analysis

Plasma samples for pharmacokinetic (PK) analyses were collected from the ewe at end of infusion and each lamb prior to UCO (baseline, prior to caffeine infusion, pre-UCO), as well as at the end of drug infusion at 1, 2, 4, 8, 24, 48, 72, 96, 120, and 144 hours following caffeine treatment. PK analysis is described in detail in Supplemental Material.

### Biochemical markers

Systemic inflammation was measured by cytokine levels 6 days after UCO as described previously^2,22,23^. We also assessed for differences in peripheral blood cells and their inflammatory ratios prior to UCO (BSN), at 8h, and on days 1, 2, 3, 5 and 6. Toxicity was assessed by measuring liver and kidney function markers. See Supplemental Material.

### Immunohistochemistry and image analysis

Histological changes in the brain were assessed at 6 days after the UCO by measuring qualitative and morphological changes in gray and white matter structures, as described previously.^22^ We examined 3 regions of white matter: periventricular white matter (PVWM), subcortical white matter located in the cingulate and first parasagittal gyrus (SCWM1 and SCWM2). Additionally, we assessed 6 regions of gray matter: cortex of the cingulate and first parasagittal gyrus (Ctx1 and Ctx2), caudate (Caud), putamen (Put), and areas within the hippocampus (Ca1/2 and Ca3).

### Neurological outcomes assessments

Our neurobehavioral assessment followed observations in sheep post-birth daily for 6 days (and included evaluation of motor function, feeding and activity at rest and their sum as the composite score (Tab.S1)^2,22,23^. Lambs without impairment achieved a maximum score of 6, while severely impaired animals with spastic paralysis, encephalopathy and an inability feed, scored 0. See Supplemental Material for additional details.

### Statistical analysis

A comprehensive description of our statistical methods^22,24,25^ is provided in Supplemental Material. We utilized an adaptive pre-clinical trial design, where high dose caffeine reached futility after only n=8 lambs. All data are shown as mean ± standard error of measurement. Differences were considered significant at *p* < 0.05.

## 3. Results

### Caffeine Pharmacokinetics

**Figure 1.**
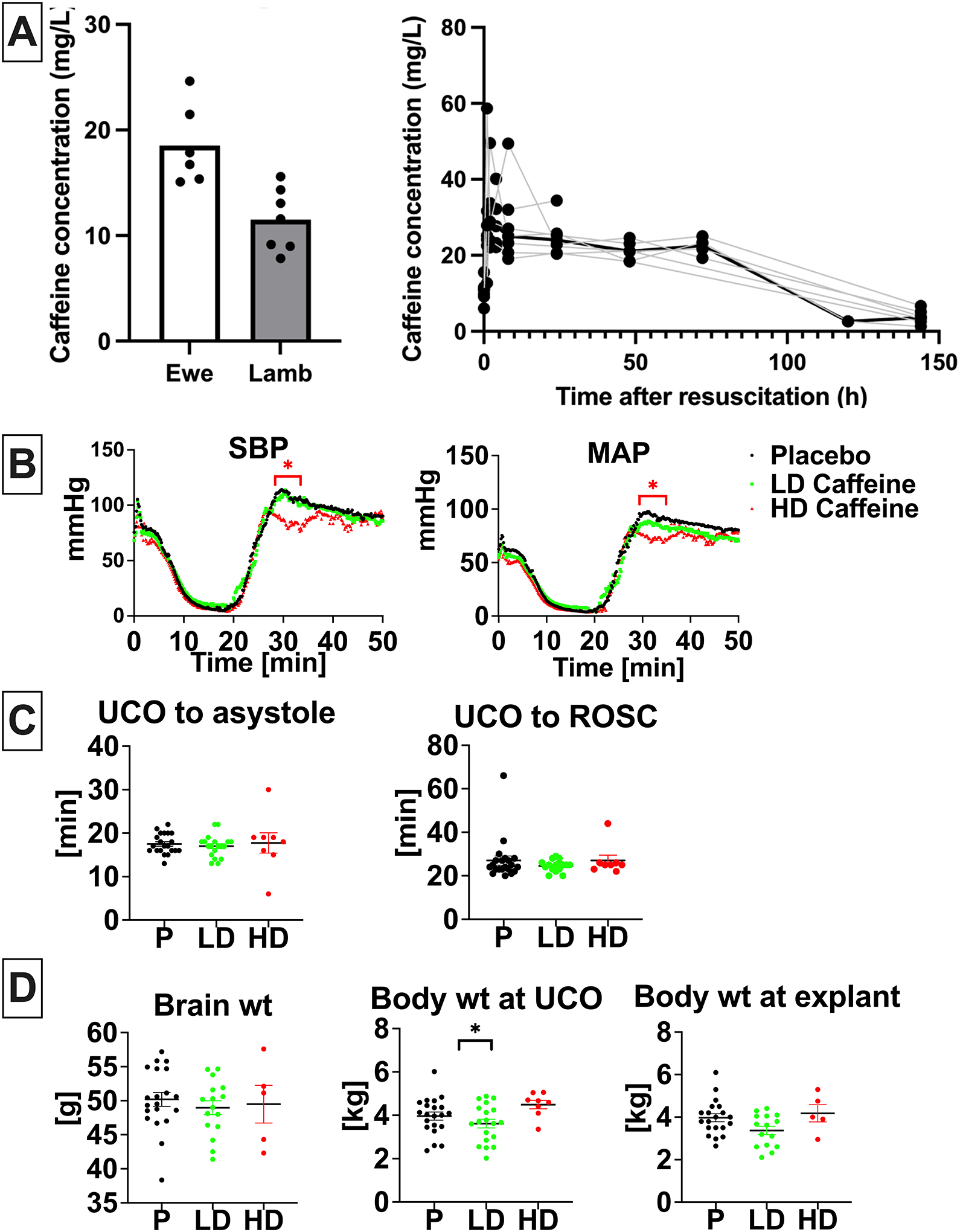
Caffeine PK and effects on resuscitation. **A,** Caffeine concentration in ewe (n=6) and lamb (n=7) plasma at the time of umbilical cord occlusion and caffeine concentration in lamb (n=7) plasma over time. **B**, Hemodynamic changes in response to UCO with drop in SBP and MAP followed by increase reflecting ROSC. Hemodynamic data was analyzed using grouped analysis of the individual group’s means for a specific time point. Placebo- n=19, LD-caffeine, n=14, HD-caffeine, n=7. **C**, Time to asystole and time to ROSC was similar among compared groups. Groups were compared using Kruskal-Wallis test. Placebo- n=21, LD-caffeine, n=18–19, HD-caffeine, n=8. **D,** Selected anthropometric parameters between the studied groups. Brain and body weight differences were assessed using ANOVA. Data shown in the graph **B** are shown as mean, graph **C** and **D** as mean ± SEM, graph. Placebo- n=20–21, LD-caffeine, n=16–19, HD-caffeine, n=5–8. UCO-umbilical cord occlusion, ROSC-return of spontaneous circulation, SBP-systolic blood pressure, MAP-mean arterial pressure, wt- weight,; LD-caffeine-treated group, green, HD-caffeine treated group- red, Placebo- black. *p<0.05.

There was highly efficient and rapid (within one hour) placental transfer of caffeine from ewe to lamb with mean percent transfer 65.5% (range 36.5%−84.9%). Maximal plasma caffeine concentration (C_max_) of 34.8±14.0 mg/L occurred after the loading dose administered to the lamb (time to maximal concentration, T_max_, 0.33±0.51 hours after infusion) and was slowly eliminated over the six-day observation period with a mean plasma half-life of 96.3±58.8 hours and an area under the curve (AUC) of 2507±382 mg*hr/L (Fig. 1A).

### Physiological outcomes

Hemodynamic parameters exhibited a similar decline following UCO in all groups (Fig.1B). The onset of ROSC following cardiopulmonary resuscitation (CPR) was also similar between all groups (Fig. 1C). HD-caffeine -treated animals exhibited lower blood pressure levels compared to placebo during the loading dose caffeine infusion after ROSC (p< 0.01–0.03), (Fig.1B). Epinephrine was administered to all animals in both groups with most animals requiring only one dose (p=0.42), (Fig.S1A). HD lambs had lower glucose vs placebo (p=0.01), however did not differ from the LD lambs (p=0.20) (Fig. S1A). HD-caffeine group experienced increased mortality (p=0.05; RR=4.594 with 95%CI (1.255–14.86)) (Fig.S1B). Caffeine administration did not lead to changes in brain or body weight compared to placebo (LD caffeine: p=0.09, HD caffeine:p=0.86)(Fig.1D). Based on interim analyses of outcomes data, HD-caffeine was discontinued early as an arm in the trial.

### Adverse events

HD-caffeine lambs experienced higher mortality within 2 days after UCO (4/8) (Fig.S1B). No other adverse events were noted. The early mortality prevented us from harvesting the brains for histological assessment, collect samples and assess neurological outcomes past day of life 2, thus these data were excluded from final analyses.

### Biochemical parameters

**Figure 2.**
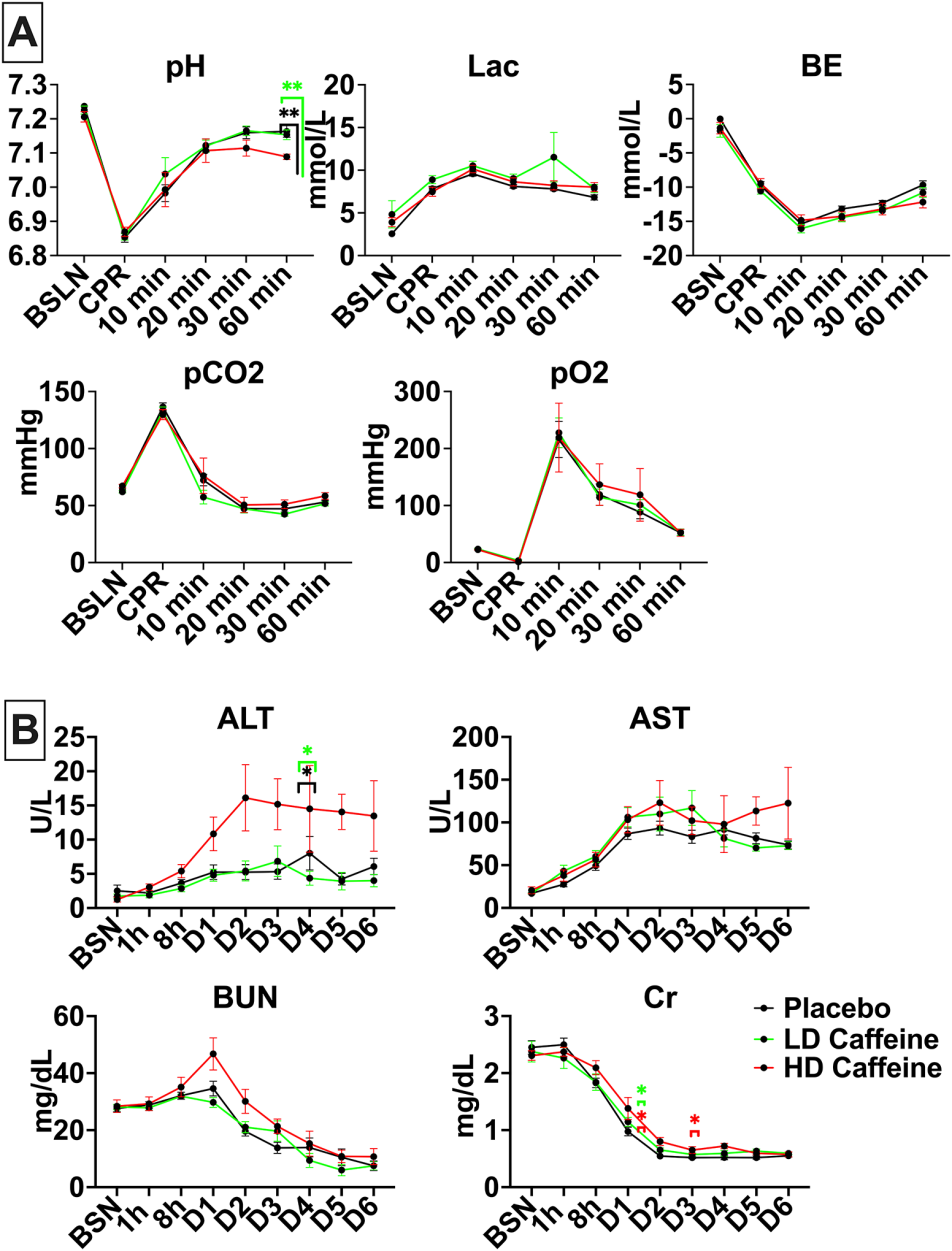
Biochemical parameters in treated vs placebo groups: **2A**, UCO leads to profound acidosis in all groups. HD-caffeine treated lambs demonstrated greater acidosis at 60 min compared to the LD-caffeine and placebo groups. All studied groups exhibited similar hyperlactatemia, base excess, carbon dioxide and oxygen levels. **2B**, HD-caffeine demonstrated significant toxicity evidenced by elevated ALT levels on day 5, as well as creatinine on day 2 and 4. No differences in AST and BUN levels were noted between the studied groups. The data was analyzed using Mixed-effect analysis with Tukey’s correction for multiple comparisons. Data in the graphs represent mean ± SEM. For 2A: HD-caffeine: n=5–8, LD-caffeine: n=19–20, Placebo: n=21; for 2B: HD-caffeine: n=4–5, LD-caffeine: n=5–15, Placebo: n=7–20. *BSN-preUCO-baseline; CPR- cardiopulmonary resuscitation; BE-base excess; lac-lactate, BUN-blood urea nitrogen, Cr- creatinine, AST- aspartate aminotransferase, ALT- alanine transaminase. LD-caffeine-treated group, green, HD-caffeine treated group- red, Placebo- black*. *p<0.05, **p<0.01.

Consistent with our prior studies^2,22,23^ the UCO protocol produced a clinically significant combined metabolic and respiratory acidosis (Fig.2A). The HD-caffeine treated lambs were more acidemic 60 min after CPR compared with placebo (p=0.0014) as well as LD-caffeine treatment groups (p=0.0015) as evidenced by lower pH values. There were no significant changes in oxygenation and ventilation among the studied groups (p>0.05). LD-caffeine did not exhibit noticeable toxicity, as reflected by similar chemistry panels relative to placebo treated animals (Fig.2B). LD-animals exhibited slightly higher creatinine levels vs placebo on day 2 (p=0.03). HD-caffeine treated lambs demonstrated elevated alanine transaminase (ALT) levels compared to LD-caffeine treatment group and placebo that reached significance on day 5 (p=0.02 and p=0.03, Fig.2B) and creatinine levels on day 2 and 4 compared to placebo (p=0.04 and p=0.04, Fig.2B).

### Inflammation

**Figure 3.**
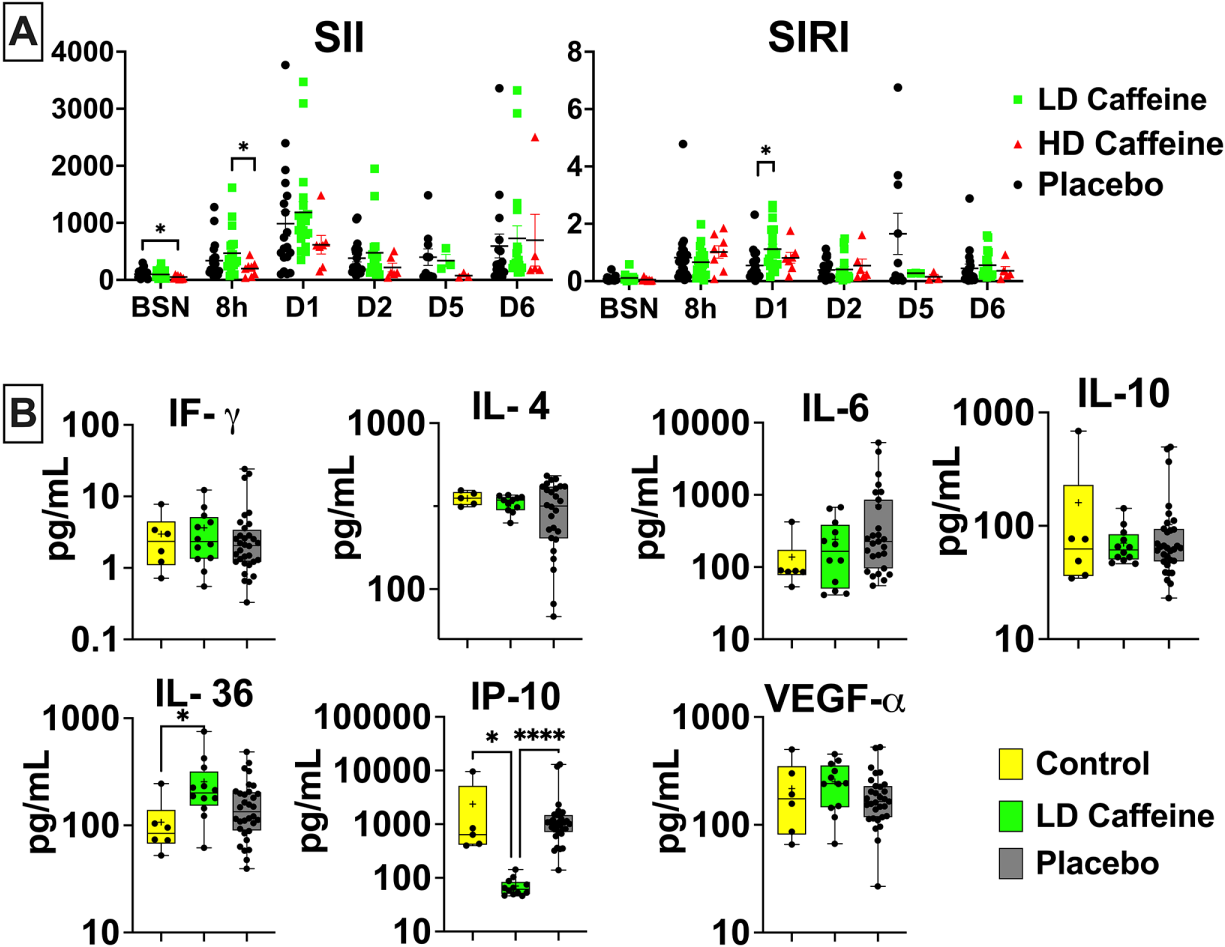
Peripheral markers of inflammation: **A,** The peripheral blood cell index SII was suppressed in HD-caffeine at baseline compared to placebo, and at 8h after UCO compared to LD-caffeine group. SIRI was elevated in the LD-caffeine group compared to the placebo on day 1. The SII and SIRI scores were evaluated by Mixed effect analysis with Tukey’s correction for multiple comparisons. The summary column graphs are showing means ± SEM. HD-caffeine: n=3–8, LD-caffeine: n=3–19, Placebo: n=10–21;**B**, At 6 days after the UCO, we measured changes in IP-10 in the LD-caffeine group compared to the placebo, as well as age-matched control and in IL-36 that was higher in the LD-caffeine group compared to control. No changes were observed in IL-4,IL-6, IFγ, IP-10, or VEGF-α. We used Mann-Whitney test. LD-caffeine: n=11–12, Placebo: n=26–32. The box graphs show mean as plus sign “+” and median with IQR. *WBC- white blood cells, ALC- absolute lymphocyte count, ANC- absolute neutrophil count, Mono- monocytes, Eos- eosinophils, PLT- platelets, SIRI- systemic inflammation response index (SIRI*=ANC*Mono/ALC), SII- systemic immune-inflammation index (SII=ANC*PLT/ALC), *p<0.05, ****p<0.0001. *LD-caffeine-treated group, green, Control- yellow, Placebo- black*.

We assessed basic markers of inflammation including white blood cell (WBC) count with differential and select cytokine levels. HD-caffeine treatment resulted in lower platelets (PLT) count compared to the LD-caffeine group 8h after UCO (p=0.004) that resolved over time (Fig.S2). The HD-caffeine group had slightly decreased systemic immune inflammation index (SII) score at baseline compared to the placebo (p=0.04) and at 8h compared to the LD-caffeine group (p=0.03) (Fig.3A). The system inflammation response index (SIRI) score was elevated on day 1 in the LD-caffeine group (p=0.01) (see Suppl Material). We did not observe changes in other subgroups of peripheral blood cells (Fig.S2). Interestingly, we detected significantly lower levels of IP-10 in LD-caffeine treated lambs versus placebo (p=0.0001) and controls (p=0.02). The levels of IL-36 were slightly elevated in the LD-caffeine group compared to controls (p=0.03), but not compared to placebo treated lambs (Fig. 3B).

### Neurohistopathological outcomes

#### Gray matter injury

**Figure 4:**
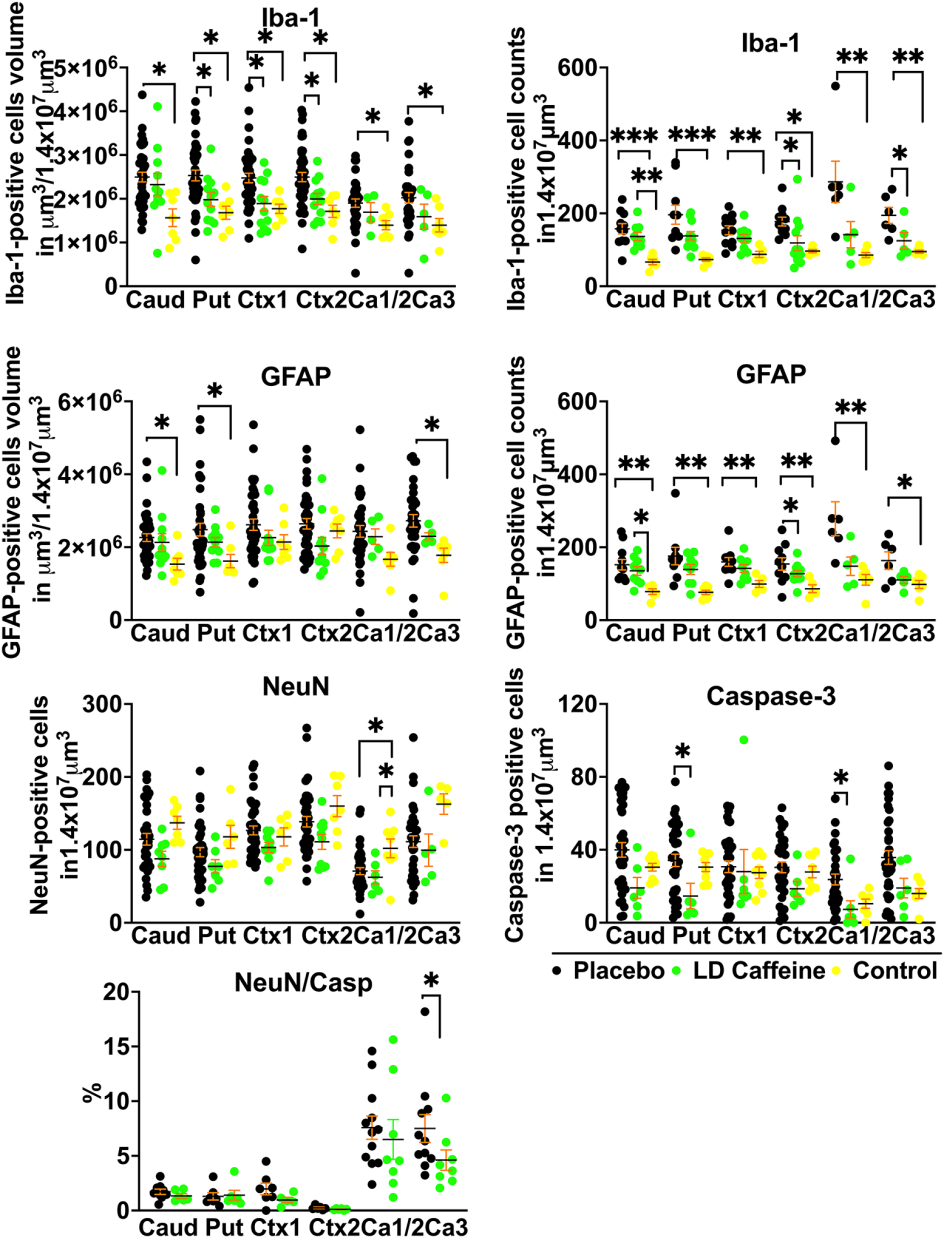
Histological changes in gray matter. **A**, We compared quantitative changes in inflammatory markers of gliosis (GFAP) and microglial accumulation (Iba-1); neuronal counts (NeuN), cellular death markers (Casp-3) in cingulate gyrus Ctx (Ctx1), 1st parasagittal gyrus Ctx (Ctx2), caudate (Caud), putamen (Put), and Ca1/2 (Ca1/2) & Ca3 (Ca3) of the hippocampus. Placebo animals (n=33–41) were compared to the LD-caffeine treated animals (n=7–11) and controls (n=4–9) using ANOVA or Kruskal-Wallis test as appropriate. Data are presented as mean ± SEM. Brackets show significance as follows: *p < 0.05. *LD-caffeine-treated group, green, Control- yellow, Placebo- black*.

We identified significant modification of gray matter injury patterns in LD-caffeine treated lambs via histological analysis at day 6. GFAP-positive astrocyte volumes increased following UCO in caudate, putamen and Ca3 region of the hippocampus that was not affected by LD-caffeine treatment (p=0.82, >p=0.99 and p=0.58). GFAP cell counts were elevated in all regions in placebo vs control (Caud: p=0.0016, Put: 0.001, Ctx1: p=0.008, Ctx2: p=0.01, Ca1/2: p=0.007, Ca3: p=0.03) and in Caud and Put (p=0.01 and p=0.02) in the LD caffeine group. In contrast, UCO resulted in significant Iba1-positive microglial accumulation in all gray matter regions evaluated which demonstrated marked region-specific reductions following LD-caffeine treatment in Put, Ctx1 and Ctx2 (p=0.047, p=0.018 and p=0.04). Similarly, Iba-1 cell counts were elevated in placebo in all regions, while LD-caffeine had elevated Iba-1 counts in caudate compared to controls (p=0.004) and LD caffeine treatment decreased the Iba-1 counts in Ctx2 and the Ca3 area of the hippocampus compared to placebo (p=0.01 and p=0.04).Total neuronal numbers were equally reduced in Ca1/2 region in both LD-caffeine treated as well as placebo treated UCO lambs when compared with uninjured controls (p=0.03 and p=0.02). The percentage of NeuN cells that were caspase positive was decreased in LD caffeine group in Ca3 region of the hippocampus (p=0.04). Total caspase-3-positive apoptotic cell numbers were reduced with LD-caffeine treatment following UCO in Ca1/2 (p=0.03) and putamen (p=0.04) suggesting widescale inhibition of cell death pathway activation (Fig. 4, S3).

#### White matter injury

**Figure 5:**
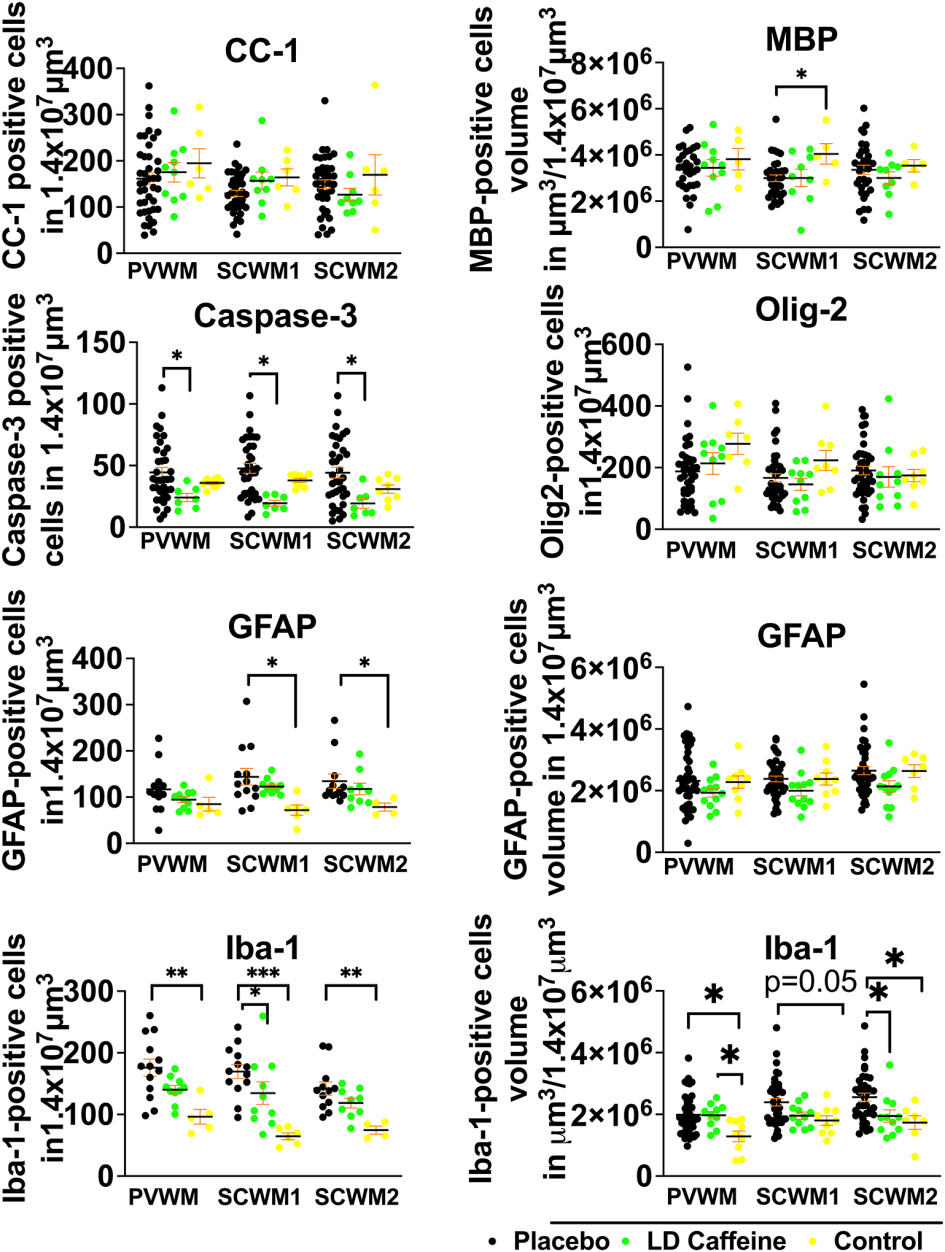
Quantitative analysis of white matter markers and markers of inflammation: While overall white matter structure was not significantly altered by UCO, there is more cell death in the placebo group compared to LD-caffeine reflected by higher number of cells labeled with cleaved caspase-3. More inflammation was noted in the SCWM2 in the placebo group reflected by accumulation of microglia and higher microglial volumes. Placebo lamb histologies (n=31–41) were compared to the LD-caffeine treated animals (n=7–12) and controls (n=5–9) using ANOVA or Kruskal-Wallis test as appropriate. Data are presented as mean ± SEM. Brackets show significance as follows: *p < 0.05. *LD-caffeine-treated group, green, Control- yellow, Placebo- black*.

LD-caffeine treatment demonstrated anti-inflammatory and anti-apoptotic effects upon histological assessment in white matter regions. No effect of UCO on GFAP-positive astrocyte volume was noted in any of the groups (placebo vs LD caffeine: PVWM, p=0.54; SCWM1, p=0.12, SCWM2: p=0.13). GFAP positive cell counts were elevated in SCWM1 and SCWM2 in the placebo group compared to control (p=0.01 and 0=0.01). The placebo treatment group demonstrated diminished MBP-stained sheets in SCWM1 following UCO when compared with controls (p=0.04) that was not affected by LD-caffeine administration (p=0.99) (Fig.5). No change was noted in oligodendrocyte precursor cells or mature oligodendrocytes in any of the groups (placebo vs LD caffeine: PVWM, p=0.75; SCWM1:p>0.99; SCWM2: p=0.99) (Fig.5A). However, we observed a higher density of microglia indicating increased inflammation in PVWM and SCWM2 following UCO compared to controls (PVWM: placebo, p=0.006; LD caffeine, p=0.04; SCWM2: placebo, p=0.02), with improvement in SCWM2 following LD-caffeine treatment (p=0.03). Iba-1 counts were elevated in placebo group in all regions compared to controls (PVWM: p=0.0018, SCWM1: p=0.0002, SCWM2: p=0.002) and LD caffeine in SCWM1 (p=0.01). Similar to gray matter regions, LD-caffeine treatment demonstrated anti-apoptotic effects, with placebo-treated animals demonstrating greater evidence of apoptotic cell death as assessed by anti-cleaved Caspase-3 immunoreactivity in all areas studied (PVWM: p=0.01, SCWM1: p=0.002, SCWM2:p=0.01) (Fig.5, Fig.S4).

### Neurobehavioral milestones

**Figure 6:**
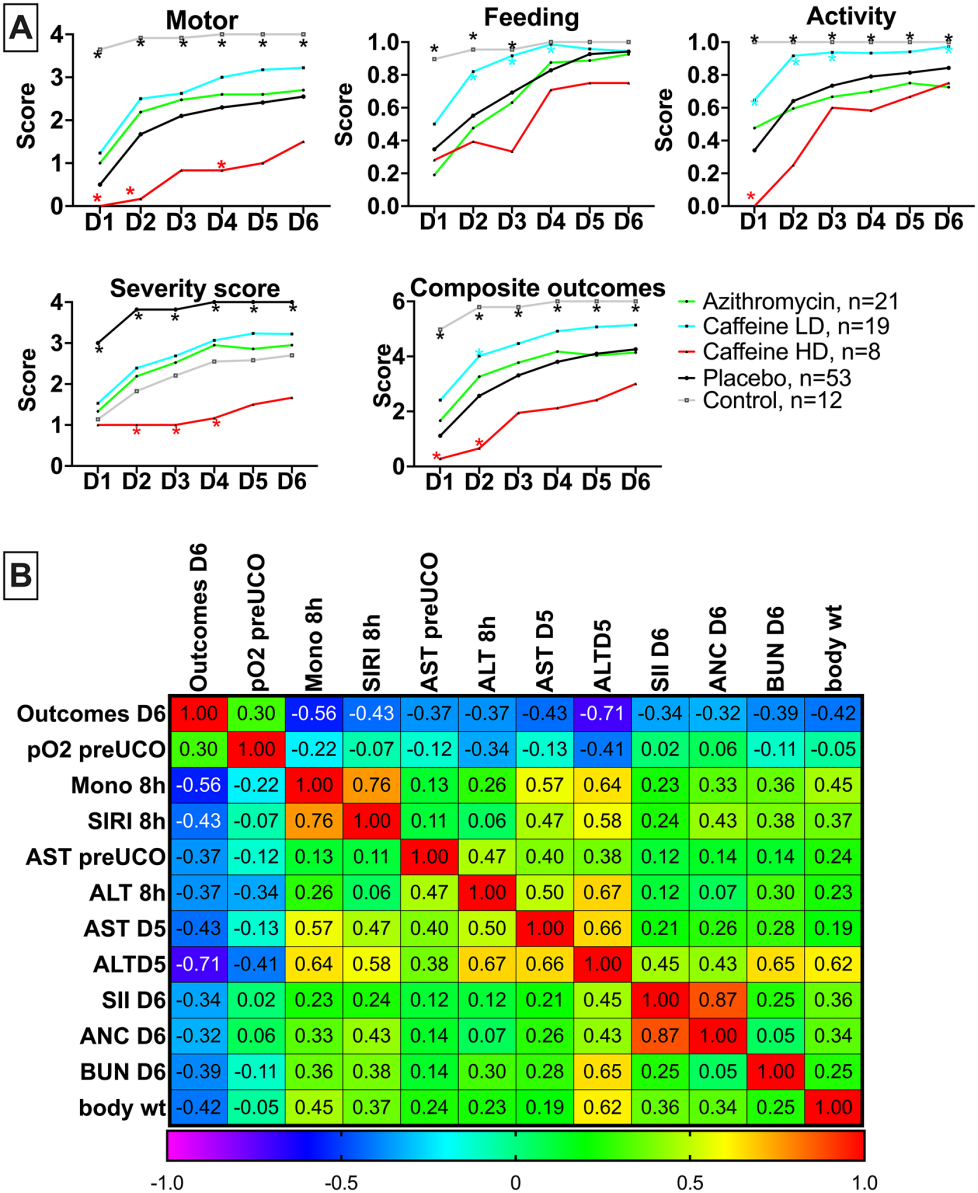
Neurodevelopmental outcomes following caffeine or azithromycin treatment: **A,** we assessed composite and individual outcomes consisting of motor function, feeding and activity and assigned a severity score. The summary graphs picture the relationship of LD- and HD-caffeine to placebo animals. *LD-caffeine-treated group, n=19, blue; HD-caffeine treated group- red, n=8; Placebo- black, n=53, Azithromycin-green, n=21, Control- purple, n=12*. **B**, The correlation matrix depicts Spearman’s correlation coefficients of selected study parameters with combined neurological outcomes scores on day 6 after UCO that reached statistical significance (p<0.05).

We compered the effects of LD- and HD- caffeine vs placebo treated animals, along with naive controls, and the animals we previously treated with azithromycin in a separate study using the identical model and endpoints^22^. LD-caffeine treated lambs scored significantly higher with respect to feeding scores on day 1–3 (p=0.002, p=0.006,p=0.003) along with activity (p=0.03, p=0.002, p=0.02) that was also improved on day 5 (p=0.03) compared with placebo-treated animals. LD-caffeine resulted in improved composite outcomes on day 2 compared to placebo controls (p=0.02) (Fig.6A, Fig.S5). HD-caffeine treatment led to worse motor outcomes compared to placebo on days 1,2 and 4 (p=0.007, p<0.0001, p=0.04), along with a worsening overall activity on day 1 (p<0.0001), worse severity score on days 2–4 (p<0.0001, p<0.0001,p<0.0001) and lower composite outcomes on day 1–2 (p=0.002, p=0.0003) when compared with placebo. Adjusting for maternal randomization and mortality, the LD-caffeine group improved motor scores on day 2 (p=0.03), total feeds+activity score on day 3(p=0.02) and severity score on day 2 after the UCO (0.04) compared to placebo (Fig.6A). These effects were more pronounced than observed following Azithromycin administration.

### Markers associated with neurological outcomes

Finally, we assessed whether any of the parameters investigated in this study were associated with poor neurological outcomes on day 6 after UCO. From the earliest parameters, worse outcomes on day 6 correlated with monocyte and SIRI levels and liver enzymes. Parameters measured at later timepoints that correlated with poor neurological outcomes included liver enzymes, SII scores, absolute neutrophil counts (ANC) and blood urea nitrogen (BUN) (Fig. 6B).

## 4. Discussion

Our results suggest that perinatal caffeine administration in a dose-limited manner is safe and may improve certain outcomes for neonates born with HIE in LMIC. The effect of caffeine on HIE in our ovine model may provide compelling evidence for its use clinically in LMIC. Caffeine is used in neonates to treat apnea of prematurity, with typical dosing of 20 mg/kg IV or orally (PO) followed by a daily maintenance dose of 5–10 mg/kg. The reported therapeutic range for this indication is 8–40 mg/L and toxicity is usually observed at levels exceeding >50 mg/L^26^. The half-life of caffeine in neonates decreases as gestational age increases and is approximately 3–4 days in neonates^27^. Due to its lipophilic properties, caffeine easily crosses the blood-brain barrier^27^. Our results confirmed that the pharmacokinetic parameters of caffeine are similar between neonatal lambs and humans and resulted in improvement in select neurologic outcomes at plasma levels within the upper end of the therapeutic range for apnea of prematurity^28^. Consistent with prior studies, we observed dose-dependent toxicity in our study^28^. Toxic effects in the HD-caffeine group were observed immediately following infusion initiation as demonstrated by reductions in blood pressure values that resolved thereafter. A bradycardic effect of caffeine has been attributed to a paradoxical induction of cholinergic activation through the inhibition of acetylcholinesterase or the ether-a-go-go (hERG) potassium channel responsible for cardiac repolarisation^29^. At higher doses, caffeine also enhances sympathomimetic effects mediated by phosphodiesterase inhibition. A caffeine-induced rise in norepinephrine and epinephrine^30^ levels could trigger a hyperadrenergic state with potential detrimental effects, including increased myocardial oxygen consumption, impaired microcirculation and heart failure after ROSC^31^. The suboptimal cardiac output associated with the HD-caffeine administration was reflected by a persistent acidosis with a lower pH at 60 min and compromised end-organ function. Although the exact cause remains unknown, the administration of HD-caffeine has been discouraged due to increased incidence of cerebellar hemorrhage^32^. Unfortunately, the limited survival of the HD-caffeine group beyond 3 days of life prevented us from gathering an adequate number of samples to measure cytokine levels on day 6 and for histological outcomes analyses.

Immune dysregulation observed during the first week after a perinatal HIE insult can be predictive of severity of neurological outcomes^33^. In our study, we detected early alterations in the inflammatory indices SIRI and SII following HIE. Elevated SIRI and SII indices are generally associated with worse outcomes after HIE^34^. Similarly, we observed a correlation of SIRI at 8 h and SII at 6 days with worse neurological outcomes. Interestingly, we found an elevated SIRI index in the LD-caffeine group on day 1 after injury and suppressed SII indices in the HD-caffeine group at baseline and 8h after UCO. We speculate that some degree of immune response activation is necessary for the clearance of damaged tissue, and in the ensuing processes of angiogenesis, tissue remodeling, and regeneration^35^.

In our analysis of cytokine levels, we found that LD-caffeine administration dramatically suppressed CXCL10 (IP-10). CXCL10 is a chemokine secreted by various immune and non‐immune cells in response to inflammatory stimuli, such as IFN‐γ^36^. Following focal stroke, CXCL10 resulted in prolonged leukocyte recruitment, astrocyte activation, and neuron sprouting^37^. CXCL10 also exacerbated blood-brain barrier injury, promoted the recruitment of NK cells to the injury site through the CXCR3 receptor and neuronal necrosis mediated by IFN-γ^36^. CXCL10 is upregulated early after brain ischemia and remains elevated for an extended period up to 10 days^38^. While in our study CXCL10 was suppressed in the caffeine group, IFN-γ levels were unchanged. This direct inhibitory effect of caffeine on the CXCL10 chemokine aligns with findings from other studies^39^. CXCL10 suppression may represent one of the potential mechanisms by which caffeine modulates neuroinflammation in the setting of neonatal HIE. We further observed increased IL-36 in the LD-caffeine group. IL-36 is both a homeostatic and inflammatory cytokine. While IL-36 is associated with neutrophilic inflammation^40^, and increased neutrophils with adverse outcomes in neonates with HIE^41^, neutrophil counts were unchanged in our studied groups. The specific role of IL-36 in neonatal HIE has not been clearly defined. Further research is needed to explore the underlying mechanisms and potential therapeutic interventions targeting immune dysregulation in the context of neonatal HIE.

Histologically, the effects of caffeine were predominantly observed in gray matter. During hypoxia-ischemia, the breakdown of extracellular ATP and the release of adenosine from ischemic cells can lead to a significant increase in extracellular adenosine levels, contributing to neuronal death^42^. Similar to findings in rodent models, our study demonstrated that LD- caffeine prevented apoptosis^43^, mostly in the Ca3 region of the hippocampus. Our observations align with the distribution of adenosine receptors, where high densities of A1 receptors are expressed in the cortex, hippocampus, and cerebellum, while A2A receptors are more abundant in the striatum and olfactory bulb^13^. Caffeine’s nonselective antagonism of A2A receptors can inhibit microglial activation and reduce cytokine release in rodent models^44^. In our study, caffeine decreased the density of activated microglial cells in the cortex of the cingulate gyrus, first parasagittal gyrus and striatum, and reduced Iba-1 counts in Ca3 of the hippocampus, which are areas of the brain consistently prone to injury in our model^2^. While in pre-term models of neonatal HIE caffeine exhibited more positive effects in white matter^45^, our study utilizing near-term lambs found less of an effect in white matter areas, although anti-apoptotic effects of LD-caffeine were consistently noted throughout all brain regions.

Caffeine has previously demonstrated beneficial effects on neurological outcomes in various animal models of neonatal HIE^9^ as well as in premature neonates^45^. Our study is the first demonstrating the feasibility, safety and efficacy of a perinatal treatment strategy designed to treat HIE in the LMIC setting where large scale studies suggest injury mechanisms occurring significantly earlier during labor and that are resistant to the beneficial effects of therapeutic hypothermia^3,7^. The therapeutic efficacy of caffeine was dose-dependent with HD-caffeine being toxic. LD-caffeine administration, however, coupled with antenatal dosing of the mother, represents a potentially promising neurotherapeutic approach that enhanced motor performance, improved feeding behavior, increased activity levels and reduced overall severity outcomes. LD-caffeine treatment when compared to azithromycin treatment showed improvement in almost all neurological outcomes by day 6.

Our study has several limitations. First, the data collected in our study represent only selected timepoints after UCO. Additional timepoints may be needed to improve our understanding of the dynamics of the neuroimmune response following UCO as the immune response to UCO is robust, with onset after initiation of CPR^46^ and alterations in systemic inflammation being detectable even in older children with cerebral palsy (CP)^47^. Thus we would suggest early timepoints, such as 1h and 4h following resuscitation, as well as later timepoints, including up to 1 year. Second, due to relatively small sample sizes, we were unable to assess sex differences, which are known to be risk factors for neurological outcomes. To detect sex differences effectively, we would need a much larger sample size of ~ 64 animals (See Supplemental Material), which is not achievable in our large animal model due to cost and ethical considerations. Lastly, we cannot exclude the possibility of subclinical seizures occurring as we monitored seizures solely based on clinical observations.

In conclusion, our study highlights the dose-dependent neuroprotective effects of perinatal caffeine administration and its positive impact on select biochemical, physiological, and histological outcomes in near-term lambs following UCO. Importantly, this treatment strategy demonstrated an excellent safety profile, making it a promising option for perinatal use in LMIC.

## Supplementary Material

Supplemental Publication Material

## Non-standard abbreviations and acronyms

HIE: hypoxic-ischemic encephalopathy
UCO: umbilical cord occlusion
ROSC: return of spontaneous circulation
CRP: cardiopulmonary resuscitation
PK: pharmacokinetics
