# Supplementary material for: Perinatal Caffeine Administration Improves Outcomes in an Ovine Model of Neonatal Hypoxia-Ischemia
Source: Stroke. 2024 Oct 21;55(11):2705–15. doi: 10.1161/STROKEAHA.124.048264 (PMC11518658; doi:10.1161/STROKEAHA.124.048264)
Supplement: Supplementary file 1 [file str-55-2705-s001.pdf]

Supplemental Material

Perinatal caffeine administration improves outcomes in an ovine model of neonatal hypoxia-ischemia

Jana Krystofova Mike, Yasmine White, Janica Ha, Ariana Iranmahboub, Cheryl Hawkins, Rachel S Hutchings, Christian Vento, Hadiya Manzoor, Aijun Wang, Brian D Goudy, Payam Vali, Satyan Lakshminrusimha, Jogarao VS Gobburu, Janel Long-Boyle, Jeffrey R Fineman, Donna M Ferriero, Emin Maltepe

- **Supplemental Methods**
- **Supplemental Figures**
- **Supplemental Tables**
- **Arrive Guidelines**

Supplemental Methods

Animals and Randomization

In this randomized, blinded efficacy study, pregnant ewes and their lambs were randomized to either caffeine or placebo. Randomization was performed at the level of the ewe. Animals in the placebo arm received an equal volume of normal saline at matched time points. Study drug was prepared by an individual separate from the research team to allow the research team to remain blinded to treatment assignment. White Dorper ewes (30 total) and their lambs of both sexes were used, total n=49 lambs. Ewes were randomly allocated to either placebo or caffeine, with lambs receiving same study drug as their mother, n= 21 placebo lambs (males=14, females=7) , low dose caffeine, (LD), n=20 lambs (males=11, females= 9). A small separate cohort of lambs received only a high-dose caffeine regimen postnatally to match the mode of delivery and pharmacological exposure in P7 rat pups that demonstrated significant neuroprotection[12]and that led to these studies, (HD), n=8 (males=5, females=3). Additional placebo animals treated with an equal volume of normal saline intravenously (IV) at matched time points, n=20-28 and naive uninjured controls, n=5-9, were pooled from prior studies, for assessment of the immunomodulatory and histological effects of caffeine. Lambs were euthanized on day 6 with an overdose of euthanasia solution (100 mg/kg pentobarbitone sodium, Lethabarb™, Virbac Pty. Ltd., Peakhurst, NSW, Australia).

Neonatal hypoxia-ischemia

Time-dated pregnant ewes were fasted for 12-24 hours prior to surgery. The ewes were induced with ketamine and propofol and anesthetized with isoflurane for surgery according to IACUC approved Standard Operating Procedure, SC-20-112, "Sheep Anesthesia: Surgical Research Facility, H-Building at TRACS". Briefly, a jugular catheter or peripheral venous catheter was placed, and the ewe given 4 mg/kg slow push IV propofol, and 1-5mg/kg Ketamine. After anesthetic induction and intubation, the ventral abdomen was shaved and cleaned. Immediately prior to surgery, the pregnant ewes underwent ultrasound imaging under general anesthesia to confirm pregnancy. After ultrasound, the ewe was then transferred into the operating room where she is placed on a mechanical ventilator. Anesthesia was maintained with 1-5 % isoflurane through the endotracheal tube. The ventral abdomen was then given a standard surgical scrub (using either Betadine or Chlorhexidine and alcohol) and the ewe placed on maintenance intravenous (IV) fluids, usually 5-15 ml/kg/hr. Oxygenation of the ewe was monitored with an O_2 saturation probe and hemodynamics were monitored with a noninvasive blood pressure cuff. A midline incision along the ventral abdomen (6-10") was made and the uterus exposed. The ewe was given IV antibiotics (penicillin G potassium 10,000-20,000 units/kg and gentamicin 1-2 mg/kg). After exteriorization of the fetal head, the fetus was intubated with an appropriately sized cuffed endotracheal tube (ETT). The lung liquid was passively drained by gravity and the ETT was plugged to prevent gas exchange during gasping. Asphyxia was induced by UCO until the onset of asystole. The umbilical cord was cut, lamb was delivered to a radiant warmer and following 5 minutes of asystole as assessed by invasive hemodynamic monitoring

the lambs were resuscitated with positive pressure ventilation with a fraction of inspired oxygen FiO_2 of 1.0. Resuscitation was not initiated with room air as asystole and the need for chest compressions is universal given the severity of the model and therefore oxygen therapy is clinically indicated. After 30 seconds of ventilation, external chest compressions were initiated. Chest compressions continued for 60 second intervals before reassessing the heart rate and these efforts continued for up to 15 minutes. Epinephrine (0.01 mg/kg) was administered intravenously if inadequate response to oxygen, ventilation and chest compressions was noted after 60 seconds of asystole following initiation of ventilation. Additional doses of epinephrine were given if animals were unresponsive to initial doses. Volume boluses were not provided as oxygen, epinephrine and chest compressions were typically sufficient to restore adequate perfusion following ROSC. ROSC was defined as sustained heart rate of 100 bpm (beats per minute) with SBP > 20 mmHg. During, as well as following resuscitation, a ventilator provided ongoing mechanical ventilation. Assisted ventilation was weaned and then discontinued when the lamb was spontaneously breathing > 50 % of the time and maintained a peripheral oxygen saturation > 85 % at an FiO_2 of 0.21. No intravenous fluids were administered during or after resuscitation. After extubation, the lambs were fed 2 oz every 4 hours by tube the first day. Afterwards, the lambs got fed 2-6 oz by bottle depending on size of lamb 4 times a day. If they were not able to bottle feed, they continued with tube feeding 2-4 oz 4 times a day with bottle attempts every day until they were euthanized. The lambs were assessed over a 6-day period to determine neurodevelopmental outcomes and euthanized on day 6 with an overdose of euthanasia solution (100 mg/kg pentobarbitone sodium, Lethabarb™, Virbac Pty. Ltd., Peakhurst, NSW, Australia).

Inclusion and exclusion criteria

Animals were allocated to experimental groups and included in the final statistical analysis following predefined inclusion and exclusion criteria. A successful UCO occlusion was confirmed during surgery using invasive hemodynamic monitoring and pulse oximetry, along with blood gas analysis. Only animals with asystole lasting > 5 min as assessed by invasive hemodynamic monitoring were included in the experiment. Death that occurred after ROSC was accounted for in neurological outcomes analyses.

Drug treatment

Caffeine citrate (caffeine) was purchased from Covertus, Inc (Portland, ME). Two caffeine dosing regimens were tested. In the first, ewes were randomized to receive either 1 g of IV caffeine citrate or placebo prior to cesarean section. Following delivery, lambs born to ewes that received caffeine were administered 20 mg/kg IV caffeine citrate over 10 minutes starting 10 minutes following resuscitation, along with two additional doses of 10 mg/kg IV caffeine citrate each at 24 and 48 hours of life (Low Dose, LD). An additional high dose (HD) caffeine arm was also investigated where no caffeine was administered to the ewe, but each lamb was randomized to receive either placebo or 60 mg/kg iv caffeine citrate following resuscitation, and 30 mg/kg iv at 24 and 48 hours of life.

Plasma PK samples were collected from each ewe immediately prior to and at the completion of caffeine infusion. PK samples were collected from each lamb prior to UCO (baseline, prior to

caffeine infusion, pre-UCO) and at 4, 8, 24, 48, 72, 96, 120, and 144 hours following caffeine treatment. Brain, liver, lung, and spleen samples were collected on day of life six for caffeine quantification Animals in the placebo arm received an equal volume of normal saline IV infusion at matched time points.

Fluid collection
Venous and arterial catheters were placed in the jugular vein and carotid artery for hemodynamic monitoring, blood sampling and drug administration. Arterial samples for blood gas analyses were taken at baseline, immediately prior to CPR (end of asphyxia, EOA), and at 10, 20, 30 and 60 min after ROSC. Samples for PK analyses and biochemical assessments of inflammation and end-organ function were drawn as stated below.

Pharmacokinetic analysis
Plasma levels of caffeine were quantified by a validated assay using liquid chromatography with tandem mass spectrometry (LC-MS/MS): To determine the caffeine concentration in lamb plasma samples, 160 μL of acetonitrile containing 25 nM internal standard and 20 μL of acetonitrile was added into 20 μL of plasma samples. The mixture was vortexed for 10 min and centrifuged at 3500 rpm for 10 min. The supernatant was transferred to autosampler vials for LC–MS/MS analysis. Tissue samples were homogenized (Precellys tissue homogenizer, Bertin Technologies, Montigny-le-Bretonneux, France) with the addition of 20 % acetonitrile in water, with a ratio of 5:1 volume (mL) to weight of tissue (g). Since no blank lamb tissues were available, the tissue homogenization was diluted 10 times with blank plasma, and then treated using the same procedure as that for the plasma samples to extract the compound for LC–MS/MS analysis. Blank plasma, the samples from un-treated control groups, were used to exclude contamination and interference. The caffeine analytical curve was constructed with 10 nonzero standards spiked in blank plasma by plotting the peak area ratio of caffeine to the internal standard versus the sample concentration. The concentration range evaluated was from 1 to 1000 ng/mL in lamb plasma. Lamb plasma caffeine concentrations (ng/mL) were determined by the LC–MS/MS method developed and validated for this study. The LC–MS/MS method consisted of a Shimadzu LC-20AD HPLC system (Kyoto, Japan), and chromatographic separation of the tested compound was achieved using a Waters XBridage reverse phase C18 column (5 cm × 2.1 mm internal diameter, packed with 3.5 μm) at 25 °C. Five microliters of the supernatant was injected. The flow rate of gradient elution was 0.4 mL/min with mobile phase A (0.1 % formic acid in purified deionized water) and mobile phase B (0.1 % formic acid in acetonitrile). An AB Sciex Qtrap 4500 mass spectrometer equipped with an electrospray ionization source (ABI-Sciex, Toronto, Canada) in the positive-ion multiple reaction monitoring mode was used for detection. Protonated molecular ions and the respective ion products were monitored at the transitions of m/z 749.5 > 591.4 for caffeineand 455.2 > 425.2 for the internal standard. We adjusted the instrument settings to maximize analytical sensitivity and specificity of detection. Data was processed with the software Analyst (v 1.6, AB SCIEX, Concord, ON, Canada).

Percentage placental transfer was calculated at each plasma PK collection time point by the following equation:

$$\% \text{ Drug Transfer} = [\text{Concentration}_{(lamb)}/\text{Concentration}_{(ewe)}] * 100$$

PK parameter estimation

Standard noncompartmental analysis was performed with R package 'NonCompart' v0.6.0 in R v4.2.0 (Bae 2022) and with PumasAI/NCA.jl v1.2.10 (Pumas-AI Inc, Baltimore, MD, USA) to provide initial estimates for PK parameters of interest including peak concentration (Cmax), half-life, and area under the concentration-time curve from dosing time to last measurement time ($AUC_{0\text{-}t}$) and area under the concentration-time curve from dosing time to infinity ($AUC_{0\text{-}\infty}$).

Neurobehavioral outcomes

We assessed the time (days) taken to reach normal lamb behavioral milestones after birth (head lift and shake; use of front and hind limbs; use of four legs; standing; walking) for a total score of 4 (Tab. S1). Ability to feed and activity at rest were evaluated separately and were reported as a sum score of 2. The severity of impairment was assessed based on the composite score of a motor function and feeding, activity. Lambs without impairment achieved a full score 6, while severely impaired animals with spastic paralysis, encephalopathy and inability to feed, scored 0.

Immunohistochemistry

Following euthanasia on day 6, brains were flushed with 500 mL of phosphate-buffered saline (PBS) and perfused with 500 mL of 4 % paraformaldehyde in 0.1 M phosphate buffer (pH 7.4). Brains were post-fixed in 4 % paraformaldehyde overnight and transferred to 20 % sucrose for 2 days and 30 % sucrose till they sank (14 days). Brains were then flash frozen in 2-methyl butane on dry ice and stored at − 80 °C. Coronal sections were cut on a cryostat (12 µm-thick serial sections). Triple immunofluorescence labeling was performed on brain sections that were defrosted and air dried at room temperature for 1 h. Following antigen retrieval in 10 mM citrate buffer (pH 6.0) for 10 min at 80 °C and a PBS wash, sections were incubated in blocking solution (5 % normal donkey serum, 0.4 % Triton X-100 in PBS) for 1 h at room temperature (RT). Primary antibody incubation was done overnight at 4 °C with rabbit anti-GFAP (GFAP, 1:500, Z0334, Agilent); mouse anti-NeuN for neurons (NeuN, 1:200, MAB377, Millipore Sigma), goat anti-Iba1 for microglia (Iba-1, 1:200, NB100-1028, Novus Biologicals), mouse anti-caspase-3 (casp-3, 1:200, NB600-1235, Novus Biologicals), goat anti-oligodendrocyte transcription factor 2 for oligodendrocytes (Olig-2,1:200 AF2418, Novus Biologicals), rat anti-myelin basic protein (MBP, 1:200, NB600-717, Novus Biologicals) and mouse anti-adenomatous polyposis coli protein clone CC-1 (CC-1, 1:100, OP800100UG, Millipore Sigma). After three 5-min PBS washes, sections were incubated for 1 h at RT with appropriate secondary antibodies: donkey anti-goat Alexa Fluor 647 (1:500, A21447, Thermo Fisher), donkey anti-mouse Alexa Fluor 568 (1:500, A10037, Thermo Fisher), donkey anti-rat Alexa Fluor 594 (1:500, A-21209, Thermofischer) and donkey anti-rabbit Alexa Fluor 488 (1:500, A21206, Thermo Fisher). For nuclear staining, sections were stained with 4',6-diamino-2-phenylindol for 5 min. Slides were then washed and coverslipped with ProLong Gold antifade (P36930, Invitrogen).

Image analysis

To define the anatomical localization of the injury, we grossly evaluated all areas of the brain on sections corresponding to s.640, and 1200 of Sheep Brain Atlas[45]at 5X magnification. For white matter injury analysis, we assessed periventricular white matter (PVWM), subcortical white matter of the cingulate and first parasagittal gyrus (SCWM1 and SCWM2). For the gray matter injury analysis, we evaluated histological changes in cortex of the cingulate and first parasagittal gyrus (Ctx1 and Ctx2), caudate (Caud), putamen (Put) and hippocampal areas of Ca1/2 and Ca3. For the final analysis, we acquired 3 confocal-like Z-stacks from the same anatomical area (25X oil objective, 10 µm thick, 1 µm Z step) using a Zeiss microscope equipped with the confocal-like optigrid device and Volocity software (version 6.3, Improvision, Perkin Elmer, Waltham, MA, USA). Every brain had a control with no primary antibodies for staining. Image capturing (using Volocity software) and analysis using Imaris software (version 9.6.2. Oxford Instruments America Inc., Pleasanton, CA) to assess NeuN, Olig-2, CC-1 and cleaved caspase-3 cells cell counts and Iba-1, GFAP, MBP volumes. Ca1/2, Ca3 NeuN- positive cells and cleaved caspase-3 cells were manually counted. The analysis was done in a blinded manner. We measured the number of cells that express NeuN, Cleaved caspase-3, Olig-2, CC-1 and total volume of cell bodies and fibers expressing Iba-1, GFAP, MBP per field of view measuring 1350 x 1050 x 10 μm^3 ($1.4x10^7$ μm^3). White matter injury was assessed by measuring the quantity and integrity of the major structural components of the myelin sheath labeled with anti-CNPase, anti-myelin basic protein, as well as by the quantity of mature oligodendrocytes stained anti-oligodendrocyte transcription factor 2 for oligodendrocytes (Olig-2) and anti-adenomatous polyposis coli protein clone, CC-1. Gray matter injury was assessed by quantifying neuronal counts stained with anti- neuronal nuclei (NeuN) for neurons. In both, gray and white matter, inflammation was assessed by quantifying the volume and cell counts of glial cells stained with anti-glial fibrillary acidic protein (GFAP), and microglial cells by staining with Iba-1- Ionized calcium-binding adaptor molecule-1 (Iba1). Apoptotic cell death was quantified by counting the total number of cleaved caspase-3 (Casp-3)-positive cells.

Biochemical markers of inflammation

We measured the cytokine levels at 6 days after the UCO using multiplex bead assay (Milliplex Ovine Cytokine/Chemokine Panel, SCYT1-91K, Millipore Sigma). 25 µL of undiluted serum samples were centrifuged at 14 000 rpm for 10 min at 4 °C. The supernatant was extracted and mixed with assay buffer and premixed beads and incubated on a plate overnight at 4 °C with agitation. After washing, the detection antibodies were added to the plate, followed by 1 h incubation at the RT. Plate was washed and analyzed on Luminex 200™ (Millipore Sigma, Merck KGaA, Darmstadt, Germany). The output data were analyzed using Belysa ™ software (version 1.1.0, Millipore Sigma, Merck KgaA, Darmstadt, Germany). We further collected complete blood count prior to the UCO (BSN), at 8h, and on days 1, 2, 5 and 6. We assessed the differences in white blood cells (WBC), neutrophils (absolute neutrophil count, ANC), lymphocytes (absolute lymphocyte count, ALC), platelets (PLT), monocytes (Mono), eosinophils (Eos). We calculated system inflammation response index (SIRI)= ANC x (Mono/ALC), systemic immune inflammation

index (SII)= PLT x (ANC/ALC), and ratios of neutrophils/lymphocytes (NLR= ANC/ALC), platelets/lymphocytes (PLR= PLT/ALC) and lymphocytes/monocytes (LMR= ALC/Mono).

Biochemical markers of toxicity

We assessed the toxicity by measuring the basic markers of end organ function. Specifically, we evaluated liver injury by measuring the levels of aspartate aminotransferase (AST), alanine transaminase (ALT) and kidney function by measuring blood urea nitrogen (BUN) and creatinine (Cr) levels.

Detailed Statistical Results

Power analysis, sample size calculation, attrition rate, blinding, and randomization

The sample size was set at 15 lambs per treatment arm, corresponding to 80 % power at detecting an 80 % relative risk reduction (RRR) in a test of two, ewe-clustered (intracluster correlation = 0.1) binomial proportions with 65 % control event and a 5 % type-I error rate (one-sided), assuming each ewe gives birth to either one or two lambs with equal probabilities. These numbers already account for the type-II error rate inflation resulting from futility testing after the first seven outcomes from each group have become available and assume 15 % attrition rate. Early stopping for futility will happen if the p-value at the interim analysis exceeds the value of 0.2822. Researchers performing experiments and analyzing data were blinded to groups. Randomization using an envelope system was applied. Envelopes were prepared and allocated in a standardized and consistent manner to avoid any potential bias or errors in the allocation process. Envelopes were prepared for each ewe in advance, each containing a slip of paper indicating which treatment group the ewe will be assigned to. The envelopes were opaque and sealed to ensure that the allocation is concealed until the moment of randomization to prevent any potential bias or influence from the participant in the allocation process. During the randomization process, the allocator of treatment selected an envelope at random, and the contents of the envelope indicated which treatment group the ewe will be assigned to. Once the ewe has been allocated to a treatment group, the allocator of treatment recorded the allocation. The study is a double-blind, as the researchers performing experiments and animal care, and data analysts are all unaware of the treatment group assignments. The researchers performing all the experiments, including the injury, post-injury care, biochemical, histological and neurological outcomes analysis were blinded to the group assignment until all measurements have been collected. The data was separated into two groups, data analysis was performed and the identity of the groups was revealed after the analysis was performed.

Statistical analysis

Analyses of biochemical, hemodynamics and histological data were performed using Prism 9 (version 9.4.1, GraphPad Software, San Diego, CA). All data are shown as mean ± standard error of measurement. Differences were considered significant at $p < 0.05$. Data was subjected to a normality test. If the data passed normality test, the differences between two groups were assessed by t-tests, otherwise we applied Mann-Whitney test. Grouped data were analyzed using one-way and two-way analysis of variance and subsequently subjected to Smidak post

hoc analyses. The data that did not pass the normality test were analyzed using Kruskal-Wallis test. The hemodynamic data was analyzed using grouped analysis of the individual group's means for a specific time point.

To analyze the effect of caffeine on the ordinal Severity Score (Tab S1), proportional odds ordinal regression models[24] were fitted at individual time points[22]. The control group consisted of the concurrent placebo arm. To incorporate fatalities in the analysis, lambs were assigned a score of '0' at the time of death and were censored thereafter. An identical approach was used for the Motor Score outcome (Tab S1), the only difference being a '-1' score being assigned to dead lambs. The treatment effects and confidence intervals reported for both these outcomes are in the log-odds-ratio scale. For the numeric Total Feeds + Activity outcome, after assignment of '-0.25' to dead lambs, a linear model for the square root-transformed outcome (with appropriate offset to ensure positivity) proved a good fit through residual diagnostics, and the reported results were subsequently the mean difference in that scale. Due to the relatively small sample size, p-values and uncertainty bounds were based on 10,000 bootstrap samples[25] rather than asymptotic. Comparisons were made between caffeine-low dose-treated (LD-Caffeine) and untreated placebo groups (Placebo). Due to the relatively small sample sizes, we were unable to assess sex differences, which are known to be risk factors for neurological outcomes.

Supplemental Figures

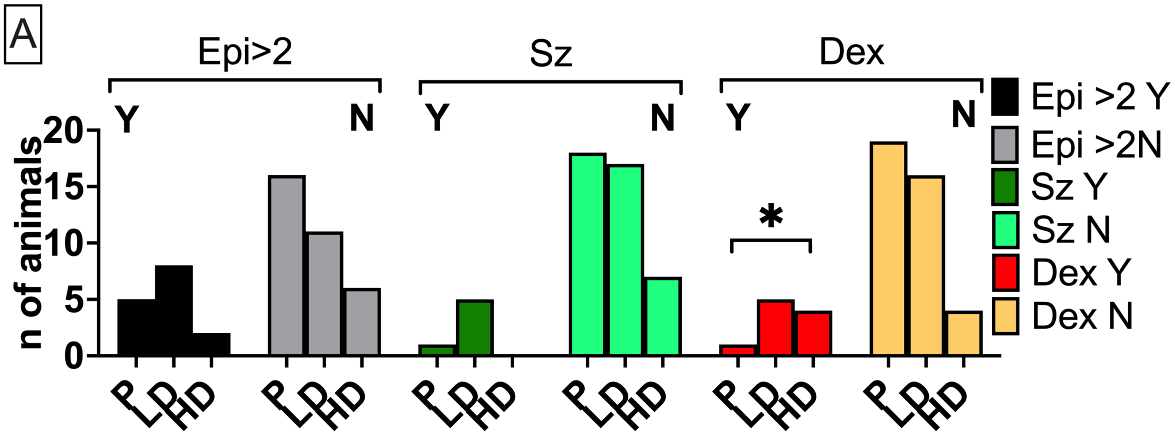

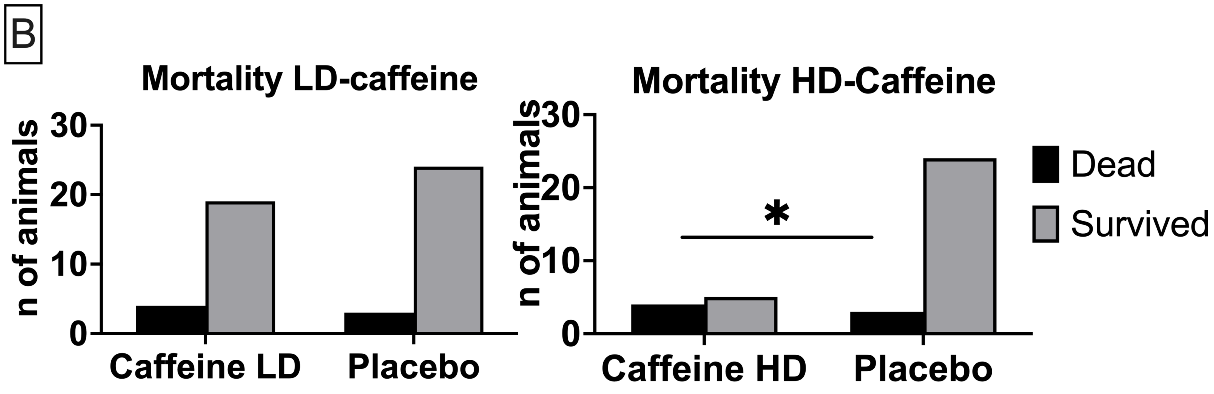

Figure S1: Resuscitation outcomes and adverse events: **A**, The incidence of 2nd dose of epinephrine was similar between the groups. Dextrose administration was higher in the HD group vs placebo, however did not differ from the LD group. The proportion of variables was assessed using Fisher's exact test. Placebo- n=19-21, LD-caffeine, n=19-22, HD-caffeine, n=7-8. **B**, HD-caffeine increased the mortality in the treated animals. **A,B** are contingency graphs Placebo- n=27, LD-caffeine, n=23, HD-caffeine, n=9.

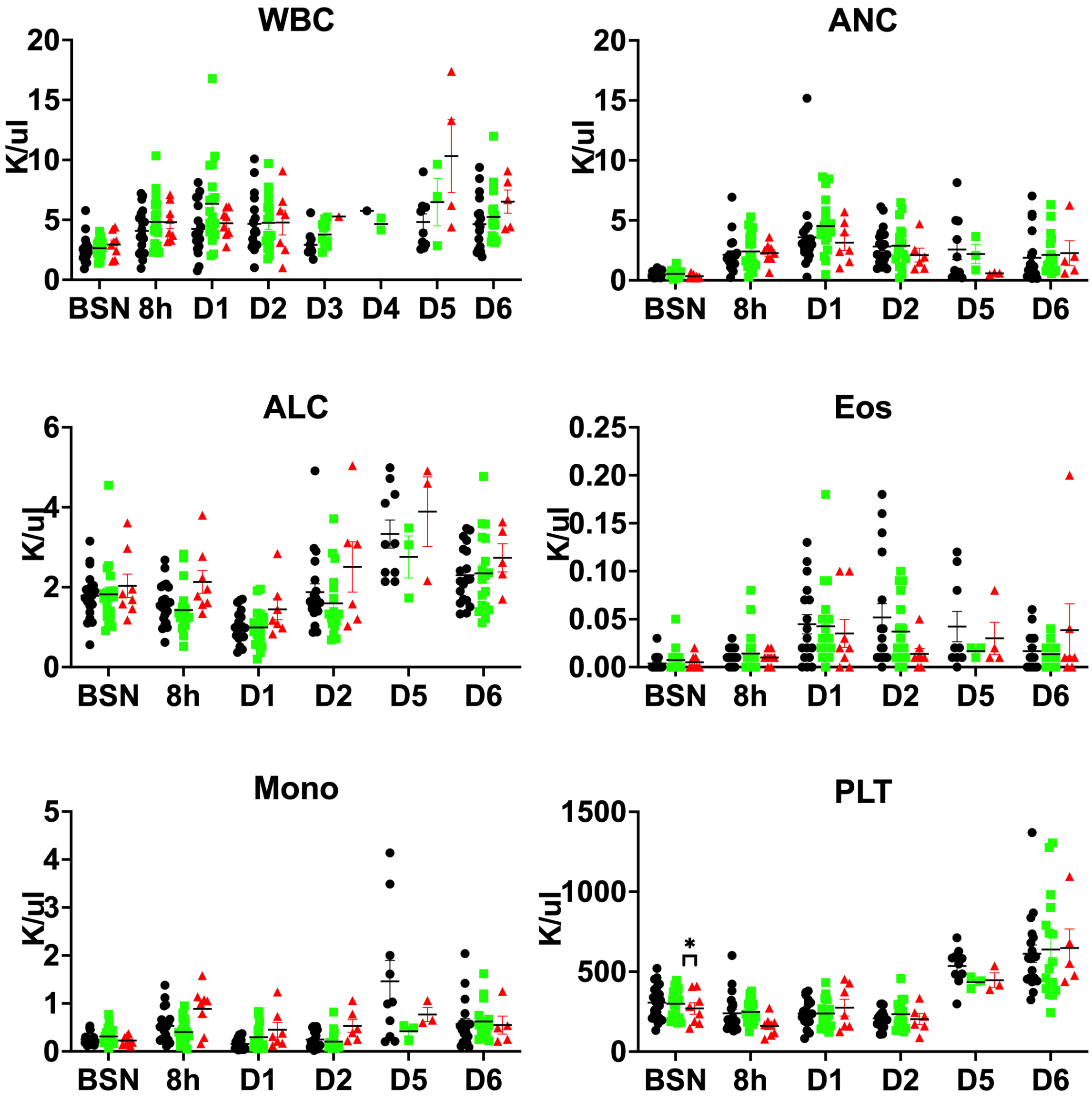

Figure S2: The cellular subgroups did not differ among HD-, LD-caffeine and placebo besides PLT counts that were lower in the HD-caffeine group at 8h after the UCO. Cellular subgroups were evaluated by Mixed effect analysis with Tukey's correction for multiple comparisons. The summary column graphs are showing means ± SEM. HD-caffeine: n=3-8, LD-caffeine: n=3-19, Placebo: n=10-21.

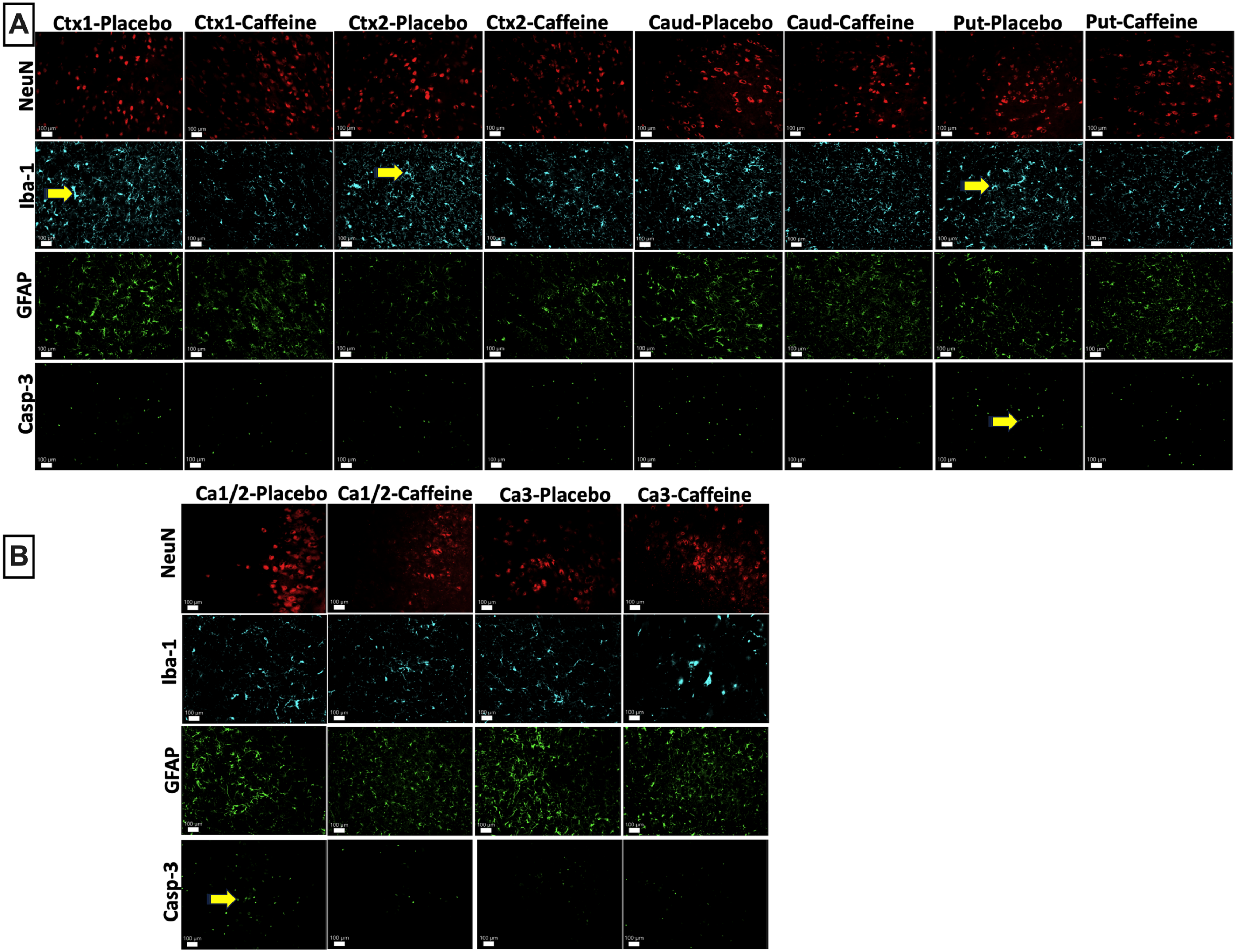

A
Ctx1-Placebo
Ctx1-Caffeine
Ctx2-Placebo
Ctx2-Caffeine
Caud-Placebo
Caud-Caffeine
Put-Placebo
Put-Caffeine
NeuN
Iba-1
GFAP
Casp-3
B
Ca1/2-Placebo
Ca1/2-Caffeine
Ca3-Placebo
Ca3-Caffeine
NeuN
Iba-1
GFAP
Casp-3

Figure S3: Histological images of gray matter: The observed quantitative changes (yellow arrow) are represented in photomicrographs by accumulation of microglial cells in Ctx-1, Ctx-2, Put (Iba-1 marker, far red) and increased cellular death (Cleaved caspase-3 marker, green) in Put and in the Ca1/2 area of the hippocampus in the placebo group. The loss of neuronal cells (NeuN, red) was notable in both groups. *GFAP-glial fibrillary acidic protein, Iba-1- Ionized calcium-binding adaptor molecule-1, Caspase-3- cleaved caspase-3, NeuN- neuronal nuclear antigen marker*.

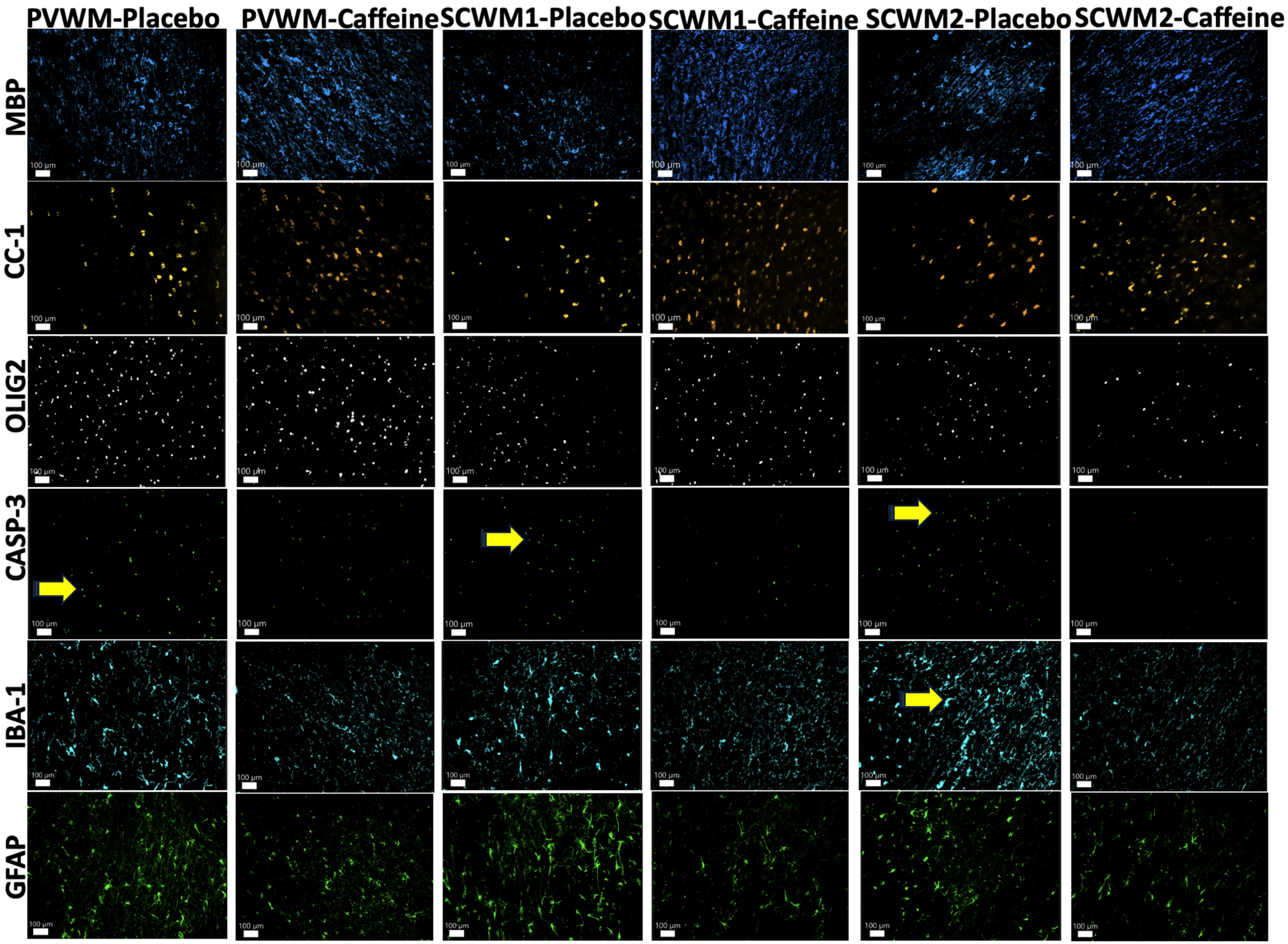
PVWM-Placebo
PVWM-Caffeine
SCWM1-Placebo
SCWM1-Caffeine
SCWM2-Placebo
SCWM2-Caffeine
MBP
CC-1
OLIG2
CASP-3
IBA-1
GFAP
100 µm

Figure S4: Histology images of white matter: representative photomicrographs of histological changes (yellow arrows) reflect increased cellular death (Casp-3) in all areas studied in the placebo groups. Microglial (Iba-1) accumulation was observed in the SCWM2 in the placebo group. *yellow- control, green- LD-caffeine, black-treated histopathology*. *GFAP-glial fibrillary acidic protein, Iba-1- Ionized calcium-binding adaptor molecule-1, Caspase-3- cleaved caspase-3, MBP- myelin basic protein, CC-1- anti-adenomatous polyposis coli clone CC-1, Olig2- oligodendrocyte transcription factor 2.*

Outcome	Day	MD or logOR [95% CI]	Pvalue
Motor Score	1	0.75 [-0.71; 2.65]	0.298
	2	1.31 [0.08; 2.88]	0.038
	3	0.81 [-0.46; 2.30]	0.214
	4	1.07 [-0.20; 2.70]	0.096
	5	1.14 [-0.11; 2.84]	0.074
	6	0.93 [-0.43; 2.46]	0.152
Total Feeds + Activity	1	0.01 [-0.23; 0.25]	0.892
	2	0.17 [-0.05; 0.40]	0.124
	3	0.19 [0.03; 0.39]	0.026
	4	0.09 [-0.03; 0.26]	0.096
	5	0.07 [-0.08; 0.23]	0.248
	6	0.06 [-0.12; 0.21]	0.512
Severity Score	1	0.35 [-1.56; 2.51]	0.656
	2	1.30 [0.02; 3.05]	0.046
	3	1.00 [-0.38; 2.70]	0.144
	4	1.07 [-0.23; 2.70]	0.104
	5	1.13 [-0.19; 2.85]	0.086
	6	0.88 [-0.47; 2.38]	0.186

Figure S5: Neurodevelopmental outcomes: The figure represents neurodevelopmental outcomes of LD-caffeine animals vs placebo. The results are reported on a logarithmic scale. Data are presented as mean ± SEM. Highlighted green is $p < 0.05$ that was considered significant. *LD-caffeine-treated group, n=19; Placebo-, n=53.*

Supplemental Tables

Table S1: Neurobehavioral assessment score:

Function	Neurological Milestone	Score
**Motor function**	**Walking**<br>**Standing**<br>**Four limbs**<br>**Front/hind lims**<br>**No movement/Spastic**	**4**<br>**3**<br>**2**<br>**1**<br>**0**
**Feeding**	**Nurses normally**<br>**Suckling well once finds the bottle**<br>**Requires assistance to find bottle; a few good suckles Minimal suckle, tube fed**	**1**<br>**0.5**<br>**0.25**<br>**0**
**Activity at rest**	**Lifts the head up, alert active**<br>**Wakes up with stimulation, attempts to lift the head Sleepy; no head lift with stimulation**	**1**<br>**0.5**<br>**0**
**Severity**	**Normal**	**4 motor + 1 feeding + 1 activity**
	**Mild**	**3 motor +1 feeding + 1 activity or 4 motor + 1 feeding/activity**
	**Moderate**	**2 motor + 1 feeding + 1 activity or 3 motor + 1 feeding/activity**
	**Severe**	**0-1 motor+1 feeding + 1 activity or 2 motor + 1 feeding/activity**

The severity of impairment was classified based on a composite score of motor function, feeding and activity. The highest score represents no impairment of the selected neurological function.

The ARRIVE guidelines 2.0: author checklist

The ARRIVE Essential 10

These items are the basic minimum to include in a manuscript. Without this information, readers and reviewers cannot assess the reliability of the findings.

Item		Recommendation	Section/line number, or reason for not reporting
**Study design**	1	For each experiment, provide brief details of study design including:<br>a. The groups being compared, including control groups. If no control group has been used, the rationale should be stated.<br>b. The experimental unit (e.g. a single animal, litter, or cage of animals).	1a caffeine-treated vs placebo vs controls<br>1b single animal
**Sample size**	2	a. Specify the exact number of experimental units allocated to each group, and the total number in each experiment. Also indicate the total number of animals used.<br>b. Explain how the sample size was decided. Provide details of any *a priori* sample size calculation, if done.	Total number and number in each group were described in Methods and Figure legends.<br>Sample size is defined in detail in Supplemental Material under Detailed Statistical Analysis
**Inclusion and exclusion criteria**	3	a. Describe any criteria used for including and excluding animals (or experimental units) during the experiment, and data points during the analysis. Specify if these criteria were established *a priori*. If no criteria were set, state this explicitly.<br>b. For each experimental group, report any animals, experimental units or data points not included in the analysis and explain why. If there were no exclusions, state so.<br>c. For each analysis, report the exact value of *n* in each experimental group.	3a Inclusion and exclusion criteria are defined in Supplemental Methods<br>3b Number of successful animals is reported in figure legends<br>3c Exact number is described in the figure legends
**Randomisation**	4	a. State whether randomisation was used to allocate experimental units to control and treatment groups. If done, provide the method used to generate the randomisation sequence.<br>b. Describe the strategy used to minimise potential confounders such as the order of treatments and measurements, or animal/cage location. If confounders were not controlled, state this explicitly.	4a Lambs were randomly assigned to treatment vs placebo group. Investigators performing the experiments and data analysis were blinded to the experimental groups.<br>4bAnimals from different groups were housed in the same room to minimize the confounders prior and after the experiment. If applicable, the samples were processed in bulks under the same conditions.
**Blinding**	5	Describe who was aware of the group allocation at the different stages of the experiment (during the allocation, the conduct of the experiment, the outcome assessment, and the data analysis).	Investigators performing the experiments and analyzing data were blinded to experimental groups. Experimental key was held by an individual not involved in the experiment and was not shared until the data was analyzed.
**Outcome measures**	6	a. Clearly define all outcome measures assessed (e.g. cell death, molecular markers, or behavioural changes).<br>b. For hypothesis-testing studies, specify the primary outcome measure, i.e. the outcome measure that was used to determine the sample size.	All measurements are defined in the methods and results.<br>As described in Methods and Results, the primary outcomes measured were improvement in histological and neurological outcomes.
**Statistical methods**	7	a. Provide details of the statistical methods used for each analysis, including software used.<br>b. Describe any methods used to assess whether the data met the assumptions of the statistical approach, and what was done if the assumptions were not met.	7a,b Statistics and statistical approach to assumptions was described in Methods and Supplemental Material

**Experimental animals**	8	a. Provide species-appropriate details of the animals used, including species, strain and substrain, sex, age or developmental stage, and, if relevant, weight.<br>b. Provide further relevant information on the provenance of animals, health/immune status, genetic modification status, genotype, and any previous procedures.	Species, sex, age was described in Methods
**Experimental procedures**	9	For each experimental group, including controls, describe the procedures in enough detail to allow others to replicate them, including:<br>a. What was done, how it was done and what was used.<br>b. When and how often.<br>c. Where (including detail of any acclimatisation periods).<br>d. Why (provide rationale for procedures).	The procedures were described in detail in Methods and Supplemental Material
**Results**	10	For each experiment conducted, including independent replications, report:<br>a. Summary/descriptive statistics for each experimental group, with a measure of variability where applicable (e.g. mean and SD, or median and range).<br>b. If applicable, the effect size with a confidence interval.	All results and statistics are described in Results, under Figure legends and in Supplemental material in detail.
